# Augmentation of Rotator Cuff Repair With Soft Tissue Scaffolds

**DOI:** 10.1177/2325967115587495

**Published:** 2015-06-10

**Authors:** Tanujan Thangarajah, Catherine J. Pendegrass, Shirin Shahbazi, Simon Lambert, Susan Alexander, Gordon W. Blunn

**Affiliations:** † John Scales Centre for Biomedical Engineering, Institute of Orthopaedics and Musculoskeletal Science, Division of Surgery and Interventional Science, University College London, Royal National Orthopaedic Hospital Trust, Middlesex, UK.; ‡ Shoulder and Elbow Service, Royal National Orthopaedic Hospital, Stanmore, UK.; *Investigation performed at the John Scales Centre for Biomedical Engineering, The Royal National Orthopaedic Hospital, London, UK*

**Keywords:** rotator cuff, tendon injuries, tissue engineering, tissue scaffolds, shoulder pain

## Abstract

**Background:**

Tears of the rotator cuff are one of the most common tendon disorders. Treatment often includes surgical repair, but the rate of failure to gain or maintain healing has been reported to be as high as 94%. This has been substantially attributed to the inadequate capacity of tendon to heal once damaged, particularly to bone at the enthesis. A number of strategies have been developed to improve tendon-bone healing, tendon-tendon healing, and tendon regeneration. Scaffolds have received considerable attention for replacement, reconstruction, or reinforcement of tendon defects but may not possess situation-specific or durable mechanical and biological characteristics.

**Purpose:**

To provide an overview of the biology of tendon-bone healing and the current scaffolds used to augment rotator cuff repairs.

**Study Design:**

Systematic review; Level of evidence, 4.

**Methods:**

A preliminary literature search of MEDLINE and Embase databases was performed using the terms *rotator cuff scaffolds*, *rotator cuff augmentation*, *allografts for rotator cuff repair*, *xenografts for rotator cuff repair*, and *synthetic grafts for rotator cuff repair*.

**Results:**

The search identified 438 unique articles. Of these, 214 articles were irrelevant to the topic and were therefore excluded. This left a total of 224 studies that were suitable for analysis.

**Conclusion:**

A number of novel biomaterials have been developed into biologically and mechanically favorable scaffolds. Few clinical trials have examined their effect on tendon-bone healing in well-designed, long-term follow-up studies with appropriate control groups. While there is still considerable work to be done before scaffolds are introduced into routine clinical practice, there does appear to be a clear indication for their use as an interpositional graft for large and massive retracted rotator cuff tears and when repairing a poor-quality degenerative tendon.

Rotator cuff tears affect 30% to 50% of patients older than 50 years and are a common cause of function-limiting pain and weakness of the shoulder.^29,61^ Many patients choose to have surgery due to disabling or progressive symptoms, and this has been reflected in a 500% increase in the rate of repair since 2001.^25^ In the United States, an estimated 75,000 rotator cuff surgeries are performed annually, and this number is likely to increase given an aging population with greater functional demands.^54^

Understanding the pathoanatomy of rotator cuff tears has improved over the past decade, and treatment strategies have evolved considerably. Poor biological healing is still problematic, with failure of tendon-bone fixation occurring in up to 26% of small to medium tears and up to 94% in large and massive tears.^8,20,27,31,48^ The cause of the high retear rate is probably multifactorial in nature and may be attributed to the older age of the patient, quality of the tissue, chronicity and size of the tear, muscle atrophy, fatty infiltration, bone mineral density, and repair technique (single- vs double-row repair).^11,12,15,52^ In selected patients, mechanical and biological enhancement of the tendon-bone interface is therefore crucial to a successful outcome after surgery.^2^

A number of synthetic and natural biomaterials have been developed into scaffolds. Those derived from extracellular matrices are thought to provide an ideal chemical and structural milieu upon which tissue integration can occur. Alternatively, synthetic scaffolds are predominantly used for their mechanical stability, although more recently their function has been enhanced by the addition of several growth factors.^59,61^ Several constructs have emerged over the years, but due to concerns over adverse host reactions, poor integration, and high retear rates, none have been implemented into routine clinical practice. The ideal material should be able to meet the physiological demands of the native tendon while providing an environment that promotes host cell–mediated healing and regeneration of a functional enthesis.

This article reviews the biology of tendon-bone healing and the current scaffolds that are used for rotator cuff repair. In doing so, we will then discuss limitations in the literature and future areas of study.

## Methods

In December 2014, a preliminary literature search of MEDLINE and Embase databases was undertaken using the terms *rotator cuff augmentation*, *rotator cuff scaffolds*, *allografts for rotator cuff repair*, *xenografts for rotator cuff repair*, and *synthetic grafts for rotator cuff repair*. A single reviewer screened the results, and all articles examining rotator cuff repair using scaffolds were included.

A total of 443 articles were found (including duplicates) after searching each of the 5 search terms. After removing 5 duplicates, 438 unique articles remained. Of these, 214 articles were irrelevant to the topic and were therefore excluded. This left a total of 224 studies that were suitable for analysis.

### Rotator Cuff Healing

The rotator cuff often ruptures at its bony insertion.^25^ Surgical reattachment has continued to pose a significant challenge due to the contrasting mechanical properties of tendon and bone. The difference in stiffness between the 2 materials generates high intrinsic tissue and intercellular stresses at the adjoining interface (enthesis). The intact healthy enthesis promotes progressive transfer of force (loading) between tendon and bone through its layered architecture, which defines its mechanical behavior. The enthesis has 2 forms: direct and indirect.^57^ Indirect entheses attach to the metaphysis and diaphysis of bone by merging with periosteum via the superficial layers of tendon, supported by deeper penetrating Sharpey fibers anchoring it to the underlying bone. In contrast, a direct enthesis, such as the rotator cuff, inserts onto bone by a layered structure comprising 4 distinct tissue types. These are, in order, tendon, demineralized fibrocartilage, mineralized fibrocartilage, and bone.^14^ The principal role of the fibrocartilaginous region is to dissipate stress concentrations at the bony insertion. The demineralized component resists compression within the distal tendon, and the mineralized component resists shearing across the bone surface.^7^

Healing of the rotator cuff takes place in 3 stages. Initially, there is an inflammatory phase with subsequent removal of tissue debris by macrophages. This is followed by fibroblast infiltration and the deposition of type III collagen to form callus. Once this collagen-rich extracellular matrix has been laid down, it is remodeled causing scar contraction. The resultant tissue has a higher ratio of type III collagen to type I collagen, a property that renders it weaker and more prone to rerupture.^6,19,21,29,45^ One factor that may contribute to the formation of this scar tissue is the mechanical strain during the initial phases of healing. At birth, the rotator cuff tendon tissue is attached to the perichondrium of the cartilaginous humeral head by an immature attachment that bears no histological resemblance to the adult insertion site. This changes first at 7 days postnatally, where a fibrocartilaginous bridge is seen, and then at 56 days postnatal, where the typical 4 zones of the enthesis are visible. Since the mechanical environment changes substantially after birth, it is plausible that this is the primary agonist for change.^19^ Furthermore, studies have indicated that compressive forces lead to the production of proteins associated with fibrocartilage (eg, aggrecan) and tensile forces lead to the production of proteins associated with tendon (eg, type I collagen).^17,55^ Other factors contributing to a poor healing response include an inadequate population of undifferentiated stem cells at the healing tendon-bone interface, the presence of macrophages at the site of tissue injury, and insufficient bony ingrowth into the tendon.^6,32^ Several techniques have been used to augment healing, but few have enhanced bone incorporation into the enthesis.^30,50,51^ This step in improving healing after rotator cuff repair may be elusive.

### Augmentation With Scaffolds

Scaffolds are used in orthopaedic surgery to induce native tissue growth. In their simplest form, they may be composed of an acellular extracellular matrix acting as a tissue bridge between tendon and bone to facilitate aligned cellular growth and collagen deposition.^1^ This basic model has been developed over recent years to incorporate biological components such as stem cells and growth factors to promote regeneration of a naturally graded enthesis.^46,59^ Currently, there are 3 forms of tendon scaffolds: xenografts, allografts, and synthetic matrices (Tables 1 and 2).

**TABLE 1:** Clinical Studies Investigating Scaffolds Used for Augmentation of Rotator Cuff Repairs*^a^*

Type of Scaffold	Study	Level of Evidence	Tear Size	Exclusion of Tears With Fatty Infiltration	Sample Size	Follow-up Period (Range)	Failure Rate on USS/MRI	Functional Outcome	Adverse Events
Porcine small intestinal mucosa	Iannotti et al^27^	2 (prospective RCT)	Large and massive (≥4 cm)	No	CG: 15 AG: 15	14 mo (12-26.5 mo)	CG: 6/15 AG: 11/15	No difference between groups using PENN	AG: 3/15 postoperative inflammatory reaction
	Phipatanakul and Petersen^42^	4 (case series)	Massive tears (≥5 cm)	No	11	26 mo (14-38 mo)	5/9	Significant improvement in UCLA and ASES scores	3/11 postoperative inflammatory reaction
	Walton et al^56^	3 (case-control)	—	No	CG: 16 AG: 15	24 mo	CG: 7/12 AG: 6/10	AG had significantly less lift-off strength, and significantly less strength in internal rotation and adduction than the CG	AG: 4/10 postoperative inflammatory reaction
Porcine dermal collagen patch	Badhe et al^3^	4 (case series)	Tears ≥5 cm	No	10	4.5 y (3-5 y)	2/10	Significant improvement in Constant score	None
Porcine dermal extracellular tissue matrix	Gupta et al^24^	4 (case series)	Full-thickness supraspinatus tear with ≥5 cm retraction/full-thickness 2-tendon tear	Yes	26	32 mo (24-40 mo)	1/26	Significant improvement in ASES and SF-12 scores	None
Acellular dermal matrix	Bond et al^9^	4 (case series)	Tears that were ≥5 cm or involved 2 tendons, or both	No	16	26.7 mo (12-38 mo)	3/16	Significant improvement in UCLA and Constant scores	None
	Barber et al^5^	2 (prospective RCT)	Large (≥3 cm) 2-tendon tears	No	CG: 20 AG: 22	24 mo (12-38 mo)	CG: 9/15 AG: 3/20	AG exhibited significantly better ASES and Constant scores	None
	Gupta et al^23^	4 (case series)	Full-thickness rotator cuff tear with >5 cm retraction	Yes	24	36 mo (29-42 mo)	1/24	Significant improvement in ASES and SF-12 scores	None
Absorbable collagen and nonabsorbable polypropylene patch	Ciampi et al^13^	3 (cohort study)	Full-thickness, 2-tendon tear with <2 cm postoperative residual retraction	Advanced fatty infiltration excluded	Collagen: 49 Polypropylene: 52 CG: 51	36 mo	Collagen: 25/49 Polypropylene: 9/52 CG: 21/51	UCLA scores at 36 months were significantly higher for the polypropylene group. Elevation and strength of the polypropylene group were significantly higher than those of the other groups	None
Absorbable poly-L-lactic acid	Proctor^43^	4 (case series)	Large to massive (2 or 3 tendons) tears with ≥3 cm retraction	No	18	42 mo (35-47 mo)	3/18	Significant improvement in ASES score	None
	Lenart et al^33^	4 (case series)	Massive tear (complete detachment of at least 2 tendons)	No	16	1.5 y (1.2-1.7 y)	8/13	Significant improvement in ASES and PENN scores	None

*^a^*AG, augmentation group; ASES, American Shoulder and Elbow Surgeons; CG, control group; MRI, magnetic resonance imaging; PENN, PENN Shoulder Score; RCT, randomized controlled trial; USS, ultrasound scan; SF-12, Short Form–12; UCLA, University of California, Los Angeles.

**TABLE 2:** Animal Studies Investigating Scaffolds Used for Augmentation of Rotator Cuff Repairs

Type of Scaffold	Study	Animal Model	Host Response	Histological Data	Biomechanical Data
Porcine small intestinal submucosa and acellular porcine dermal patch	Nicholson et al^41^	Ovine, infraspinatus detachment	No adverse reaction	At 24 weeks, porcine dermal patches were integrated into adjacent tendon tissues. A more diverse tissue response was seen with small intestinal submucosa. This was characterized by the formation of ectopic bone and fibrocartilage	At 24 weeks, failure loads were identical between groups
Porcine small intestinal submucosa	Zalavras et al^60^	Rat, supraspinatus detachment and creation of a large 4-mm defect	No adverse reaction	At 16 weeks, the graft group exhibited fibroblastic ingrowth, neovascularization, and a collagenous extracellular neomatrix. In contrast, the nonaugmented group demonstrated a disorganized fibroblastic response lacking any orientation	The small intestinal submucosa group demonstrated a significantly higher ultimate force to failure than the nonaugmentation group
Acellular dermal matrix	Ide et al^28^	Rat, supraspinatus and infraspinatus detachment	No adverse reaction	Histologic incorporation of the graft into a structure resembling normal tendon at 12 weeks after surgery	Nonaugmentation group exhibited lower mean ultimate force to failure
	Adams et al^1^	Canine, infraspinatus excision	No adverse reaction	Within 6 weeks, histologic evidence of native cell infiltration and neotendon development was observed	Within 12 weeks, the strength of the dermal matrix graft repair was equivalent to that of autograft control tendon repairs
PGA sheet	Yokoya et al^59^	Rabbit, full-thickness defect of rotator cuff	No adverse reaction	In the MSC group, fibrocartilage layers and Sharpey fibers were found regularly in the insertion site at 8 weeks compared with PGA alone. A large volume of type I collagen was found in comparison with type III collagen at 16 weeks in the MSC group, whereas type III collagen was more prevalent than type I in the PGA group	At 16 weeks, regenerated tendons in the MSC group had better tensile strength than in the PGA
bFGF-loaded PLGA electrospun fibrous membrane	Zhao et al^61^	Rat, chronic rotator cuff tear model	No adverse reaction	PLGA membrane was associated with improvements in fibrocartilage and collagen organization at the healing enthesis compared with rotator cuff repair without augmentation. The bFGF-loaded PLGA membranes significantly improved collagen organization	Electrospun fibrous membrane groups had a greater ultimate load to failure and stiffness than the control group at 4 and 8 weeks. The bFGF-loaded PLGA membranes had the highest ultimate load to failure, stiffness, and stress of the healing enthesis

*^a^*bFGF, basic fibroblast growth factor; MSC, mesenchymal stem cell; PGA, polyglycolic acid; PLGA, poly(lactic-*co*-glycolic acid).

#### Xenografts

Extracellular matrices derived from either xenogenic or allogenic material are excellent 3-dimensional (3D) scaffolds for tissue engineering and can be utilized for the surgical regeneration of musculoskeletal, dermal, cardiovascular, and gastrointestinal tissues.^18^ To avoid adverse immune reactions, a rigorous decellularization process is an important step in the development of these biological scaffolds. This can be accomplished by using gamma irradiation or physical, chemical, or enzymatic techniques. Physical methods entail freezing or mechanical agitation to lyse native cells, whereas chemical-based strategies use hypotonic solutions or detergents to lyse the cells in the harvested tissue, which is then washed to remove them. Trypsin, an enzyme that hydrolyzes proteins, is found in the digestive system of vertebrates. When used as a single agent, it is capable of degrading cellular material within a matrix, but its effect can be enhanced when used in combination with gamma irradiation.^10,18^

Porcine small intestinal submucosa contains type I collagen and growth factors such as fibroblast growth factor–2 (FGF-2), transforming growth factor–β (TGF-β), and vascular endothelial growth factor (VEGF).^4,37^ It has been used as a biological scaffold that can support cellular attachment and result in angiogenesis and collagen formation, thus forming a structure similar to that of the native enthesis.^40^ Zalavras et al^60^ used porcine small intestinal mucosa to reconstruct a large supraspinatus tendon defect in a rat model. No adverse reactions were identified. At 16 weeks, the graft group demonstrated fibroblastic ingrowth, neovascularization, and a collagenous extracellular neomatrix. In contrast, the nonaugmented group exhibited a disorganized fibroblastic response. The small intestinal submucosa group also demonstrated a significantly higher ultimate force to failure than the nonaugmented group. Nicholson et al^41^ evaluated 2 commercially available xenografts (porcine acellular dermal patch and porcine small intestinal mucosa) in a sheep model of infraspinatus detachment. At 24 weeks, porcine dermal patches were integrated into adjacent tendon tissues, whereas a more diverse tissue response was seen with small intestinal submucosa. This was characterized by the formation of ectopic bone and fibrocartilage. Failure loads were identical between groups at 24 weeks.

Clinical studies evaluating the efficacy of porcine xenografts to augment rotator cuff tears have had varied results. In one of the few randomized controlled trials investigating rotator cuff healing with a xenograft, Iannotti et al^27^ treated 15 shoulders with porcine small intestinal mucosa (Restore Orthobiologic Implant; DePuy) and compared them with 15 traditional open repairs. No significant improvement in healing or functional outcome was found.

Phipatanakul and Petersen^42^ used porcine small intestinal mucosa to augment the repair of massive rotator cuff tears. Despite an improvement in functional outcome scores, only 44% of repairs were partially or completely intact postoperatively. Furthermore, 3 complications occurred, including 1 infection and 2 skin reactions. Poor results were also reported by Walton et al,^56^ who found that patients whose rotator cuffs were repaired using the Restore Orthobiologic Implant, a collagen-based material derived from the small intestinal mucosa of pigs, had decreased muscle strength, greater impingement in external rotation, slower rate of pain resolution, and reduced participation in sport. Two years postoperatively, magnetic resonance imaging demonstrated comparable retear rates between the study group and nonaugmented controls. Because of the high number of severe inflammatory reactions that required further surgery, use of this implant was discouraged. Malcarney et al^34^ also described this reaction in a series of 4 cases, and Zheng et al^62^ reported that it may be due to residual porcine cellular elements in the graft.

Porcine dermal collagen has been shown to support fibroblast infiltration and revascularization and has been used in the reconstruction of human soft connective tissue defects where loss of the dermis has occurred. Badhe et al^3^ reported a series of 10 patients in whom a porcine dermal collagen patch was incorporated in open repairs of extensive rotator cuff tears. These tears were at least 5 cm in size and involved both the supraspinatus and infraspinatus tendons. Pain, range of movement, and functional outcome improved significantly. No adverse effects were reported, but the evidence was limited by the lack of a suitable control group. Gupta et al^24^ studied 26 patients who underwent interpositional reconstruction of 2-tendon or massive, irreparable rotator cuff tears using Conexa xenograft (Tornier Inc). At a minimum 2-year follow-up, there was improvement in range of movement and functional outcome, as assessed by the American Shoulder and Elbow Surgeons (ASES) score. One failure was noted after a fall that caused a complete tear at the graft-bone interface due to suture anchor pullout. No cases of infection, inflammatory changes, or tissue rejection were found. Comprehensive processing of the graft in addition to its noncrosslinked structure was thought to be responsible for the lack of an adverse reaction.

The current body of evidence suggests that xenografts do not appear to enhance rotator cuff repair in humans, with retear rates similar to nonaugmented controls.^27,56^ Major concerns have also been raised over their immunogenic potential and associated severe inflammatory reactions.^34,62^ This is most likely due to traces of DNA and TGF-β remaining in the graft material despite thorough decellularization. Another less commonly found cell-associated marker responsible for hyperacute rejection of porcine xenografts is galactose-α-1,3-galactose (α-Gal). The α-Gal epitope is synthesized on glycolipids and glycoproteins present in nonprimate mammals by the glycosylation enzyme α-1,3-galactosyltransferase. This epitope, is not present in humans, who instead have the anti-Gal antibody, which constitutes approximately 1% of circulating immunoglobulins and specifically targets α-Gal.^35,36,62^

#### Allografts

Allogenic matrices are produced by the decellularization of cadaveric material from humans and are capable of bridging soft tissue defects while reducing the risk of graft rejection. Ide et al^28^ used acellular dermal matrix (GraftJacket; Wright Medical Technology) in a rat model to reconstruct large rotator cuff tears and induce tendon regeneration. When compared with untreated controls, the graft group exhibited a greater mean ultimate force to failure and superior histological outcomes with fibroblastic ingrowth at the tendon-bone interface, neovascularization, and production of a collagenous extracellular matrix. Fibroblasts were also oriented along stress lines, and the graft could not be identified in any of the specimens. Adams et al^1^ compared human acellular dermal matrix graft (GraftJacket) with an autologous excised tendon used to repair a full-thickness infraspinatus tear in a canine model. Within 6 weeks of application, the dermal matrix displayed evidence of native cell infiltration and neotendon development. At 12 weeks, the strengths of the 2 repairs were comparable, and at 6 months, a remodeled tendonlike structure characterized by Sharpey fibers was visible in the graft group.

A number of human studies have also examined the effect of acellular dermal matrix on rotator cuff repair. Bond et al^9^ reviewed the outcome of 16 patients with massive, contracted, immobile rotator cuff tears that were treated with arthroscopic placement of a GraftJacket allograft. This yielded a failure rate of 19%, which is considerably lower than the 30% to 94% quoted in the literature.^29^ No complications were noted, and follow-up analysis of the graft at 12 months illustrated its viability. Furthermore, in 1 of the patients who had a documented failure, a biopsy revealed partial neotendon formation at the site of graft insertion. Histological analysis of the remaining specimens was not undertaken, and therefore, the extent of any tendon remodeling remains unknown. Barber et al^5^ further assessed the effectiveness of the GraftJacket in a randomized, prospective, multicenter clinical study of 42 patients undergoing arthroscopic repair of large (>3 cm) rotator cuff tears. At 24-month follow-up, using the ASES and Constant scores, superior functional outcomes were noted in the augmented group compared with the nonaugmented controls. Significantly more intact repairs were also found in the GraftJacket group using enhanced magnetic resonance imaging, and no adverse reactions related to the acellular human dermal matrix were observed. Similar results were found by Gupta et al,^23^ who examined 24 patients who underwent interpositional repair of massive irreparable rotator cuff tears using the GraftJacket. At a mean 3-year follow-up, range of movement and ASES scores significantly improved after surgery. Ultrasonography demonstrated fully intact repairs in 76% of the cohort, with all remaining patients having partially intact repairs. No complete tears were found.

Although the results of acellular dermal matrices are promising, there have been no large human studies conducted evaluating their effectiveness. Bony ingrowth into a healing tendon is crucial for regeneration of a functional enthesis, but little sign of this is seen with current allografts.^6^ Despite no serious complications being reported, there are some potential problems with allogenic matrices. Like xenografts, there have been concerns over the presence of residual DNA.^22^ This may cause an inflammatory response and increase tendon degeneration.^62^ It has also been shown that the elastic moduli of allografts are less than that of autogenic tendons, suggesting that they have a limited mechanical role.^16^

#### Synthetic Grafts

Because of ongoing concerns regarding the immunogenicity of both xenografts and allografts, there has been considerable interest in synthetic constructs. Degradable polyesters such as poly(lactic-*co*-glycolic) acid (PLGA), poly-l-lactic acid (PLLA), and polydioxanone (PDO) have emerged as potential biomaterials to create this novel group of implants.^25^ On their initial conception, rapidly absorbable sheets of polyglycolyic acid (PGA) were used to regenerate the enthesis; however, they displayed poor mechanical properties and created a tendon insertion comprised primarily of type III collagen.^58^

Moffat et al^39^ conducted an in vitro evaluation of well-aligned and unaligned electrospun degradable PLGA-based nanofiber scaffolds with pre-engineered mechanical properties matching those of the native tissue. Scaffolds consisting of well-aligned nanofibers were found to have a higher elastic modulus, yield strength, and ultimate strength than unaligned specimens. This precise arrangement also had a significant effect on the cellular response to the graft, with fibroblasts attaching along the long axis of the aligned nanofibers in contrast to the unaligned scaffolds where they adopted a random orientation. The process of electrospinning has also been shown to minimize the immune response compared with processing the same materials into films, making it an appealing method to produce these constructs.^47^

By incorporating various biological components into synthetic scaffolds, their functional properties have been enhanced. In a rabbit model of an infraspinatus tear, Yokoya et al^59^ found that the addition of mesenchymal stem cells to a PLGA sheet resulted in the formation of fibrocartilage and Sharpey fibers at the insertion site. The resultant enthesis also had a greater proportion of type I collagen and a better tensile strength when compared with controls without mesenchymal stem cells. Zhao et al^61^ applied basic fibroblast growth factor (bFGF)–loaded PLGA electrospun fibrous membranes to a rat model of a chronic rotator cuff tear and compared the results with conventional repairs. Local administration of the PLGA membrane was associated with improved collagen organization and greater fibrocartilage production during the initial phases of healing. This was accompanied by a significantly greater ultimate load to failure, which was maintained at progressive time points.

Few human studies examining the effect of synthetic scaffolds on regeneration of the enthesis have been conducted. In a nonrandomized retrospective 3-year follow-up study, Ciampi et al^13^ compared the results of mini–open repair of posterosuperior massive rotator cuff tears between nonaugmented controls, augmentation with an absorbable collagen patch, and augmentation with a synthetic nonabsorbable polypropylene patch. The polypropylene patch performed the best, exhibiting a significantly lower 12-month retear rate and superior functional outcome (using the University of California, Los Angeles shoulder rating scale), abduction, and elevation at 36 months. No adverse reactions were related to patch application. Proctor^43^ evaluated the functional results of 18 consecutive patients with large to massive rotator cuff tears treated with a woven mesh of absorbable poly-l-lactic acid (X-Repair; Synthasome Inc). A combination of ultrasound and magnetic resonance imaging showed that 83% of patients had intact repairs 12 months after surgery. At 42 months, 1 additional failure occurred, which reduced long-term survival to 78%. There was a progressive improvement in functional outcome in the cuff repair survivor group, assessed by the ASES scoring system, at all time points. Also investigating this scaffold, Lenart et al^33^ reviewed 16 consecutive patients with massive or recurrent rotator cuff tears that underwent open repair with the graft. At a mean of 1.5-year follow-up, the ASES and the PENN Shoulder Score significantly improved despite only 5 repairs being intact on magnetic resonance imaging.

Although many studies investigating synthetic scaffolds have yielded encouraging results, there are several concerns over the degradation products of the polymers used to produce them. High levels of lactic and glycolic acid have been shown to impair osteoblast proliferation and inhibit matrix mineralization, whereas in nontoxic concentrations, they were found to decrease cellular proliferation and increase differentiation of osteoblasts.^38^ These toxic effects have been shown to vary between polymers, and thus further research is required to ensure that the liberation of these degradation products remains within safe levels for the duration that the implant is in situ.^53^

## Discussion

Scaffolds are used to enhance healing of the rotator cuff, but little data exist to support the hypothesis that they will improve the biomechanical properties of the repair construct. Using a validated spring network model developed for investigating nonaugmented and augmented human rotator cuff repairs, Aurora et al^2^ established that the mechanical properties of the overall repair were primarily influenced by the quality of tendon-bone fixation. If this is compromised by fixation in osteopenic bone and repair of a chronic degenerative tendon, there is a concomitant reduction in the yield load (43%) and stiffness (62%) of the construct. Scaffold augmentation under these circumstances may mitigate the reduction in mechanical properties by bearing approximately 45% of the total load.^2^

After implantation, scaffolds may undergo degeneration or remodeling, but the mechanism by which these processes occur is still poorly understood, as are the long-term effects of the degradation products they may release. Small intestinal submucosa is rendered acellular during processing and exclusively consists of an extracellular matrix that is rapidly resorbed and subsequently remodeled. The resultant structure often resembles tissue that is normally found at the site of application; however, due to reports of immunogenic reactions, its use has been discouraged.^37,56^ To overcome this, a number of allogenic matrices have been manufactured from decellularized human cadaveric material. Despite no serious complications being reported, there have been concerns over the presence of residual DNA, which may cause an inflammatory response and increase tendon degeneration.^22,62^ Biodegradable synthetic scaffolds have offered an alternative to more traditional biomaterials, but they are still in their relative infancy, with no long-term studies available for analysis. They are created to degrade over time into nontoxic metabolites. Scaffolds made from nonbiodegradable polymers, although an exception, persist for the lifetime of the patient.^44^

Animal models provide a unique opportunity to study the effects of scaffolds on tendon. Even so, animal models cannot be used to accurately predict human response to biomaterials. The principal advantages of animal studies are that several comparative groups may be examined simultaneously with robust methodology, tissues may be harvested for analysis at several time points, and injuries can be reproduced consistently. However, small and large animal models do not accurately reflect the conditions within the human shoulder during tearing of the rotator cuff, since many utilize quadrupeds, which subject their tendons to different loads and entail different joint kinematics.^49^ The acute tendon injuries in these models are also different from clinical scenarios, which often entail degeneration of the rotator cuff prior to the tear.^26^

Scaffolds have received considerable attention in the literature and formulate an important part of the shoulder surgeon’s armamentarium for the treatment of complex tears of the rotator cuff. Despite a number of novel biomaterials being developed into biologically and mechanically favorable constructs, there is a paucity of clinical trials examining their effects on tendon-bone healing in well-designed, long-term follow-up studies with appropriate control groups.^5,27^ Indications for the use of scaffolds are also inconsistent between studies, and therefore, current evidence cannot be used to inform management of specific patient groups such as older individuals who are prone to poorer outcomes and tears with fatty infiltration.^52^

## Conclusion

Over the past decade, there has been an increasing trend toward operative intervention for the treatment of rotator cuff tears. With an aging population, it is likely to represent one of the most common soft tissue procedures performed in the future. To improve current surgical outcomes, it is imperative that new augmentation techniques are evaluated with long-term follow-up studies so that the precise effect of the scaffold and its degradation products can be determined. Evaluation should assess patient-reported outcomes, function, and imaging of the repair. Animal studies also have an important role in the examination of the tendon-bone interface, but models should attempt to reproduce the degree of chronicity often present in the torn rotator cuff. The literature thus far has evaluated scaffolds, growth factors, and some biologically enhanced materials, although none have been able to regenerate the mechanical properties of a normal, graded enthesis. While there is still considerable work to be done before scaffolds are introduced into routine clinical practice, there does appear to be a clear indication for their use as an interpositional graft for large and massive retracted rotator cuff tears and when repairing a poor-quality degenerative tendon.
